# A novel hybrid supervised and unsupervised hierarchical ensemble for COVID-19 cases and mortality prediction

**DOI:** 10.1038/s41598-024-60637-y

**Published:** 2024-04-29

**Authors:** Vitaliy Yakovyna, Nataliya Shakhovska, Aleksandra Szpakowska

**Affiliations:** 1 Faculty of Mathematics and Computer Science, University of Warmia and Mazury in Olsztyn, Ul. Oczapowskiego 2, 10-719 Olsztyn, Poland; 2 Artificial Intelligence Department, Lviv Polytechnic National University, 12 S. Bandery St, Lviv, 79013 Ukraine; 3 Universytet Rolniczy, 31120 Kraków, Poland

**Keywords:** COVID-19, Machine-learning, Ensemble model, Classification, Regression, Supervised learning, Unsupervised learning, Diseases, Health care

## Abstract

Though COVID-19 is no longer a pandemic but rather an endemic, the epidemiological situation related to the SARS-CoV-2 virus is developing at an alarming rate, impacting every corner of the world. The rapid escalation of the coronavirus has led to the scientific community engagement, continually seeking solutions to ensure the comfort and safety of society. Understanding the joint impact of medical and non-medical interventions on COVID-19 spread is essential for making public health decisions that control the pandemic. This paper introduces two novel hybrid machine-learning ensembles that combine supervised and unsupervised learning for COVID-19 data classification and regression. The study utilizes publicly available COVID-19 outbreak and potential predictive features in the USA dataset, which provides information related to the outbreak of COVID-19 disease in the US, including data from each of 3142 US counties from the beginning of the epidemic (January 2020) until June 2021. The developed hybrid hierarchical classifiers outperform single classification algorithms. The best-achieved performance metrics for the classification task were Accuracy = 0.912, ROC-AUC = 0.916, and F1-score = 0.916. The proposed hybrid hierarchical ensemble combining both supervised and unsupervised learning allows us to increase the accuracy of the regression task by 11% in terms of MSE, 29% in terms of the area under the ROC, and 43% in terms of the MPP metric. Thus, using the proposed approach, it is possible to predict the number of COVID-19 cases and deaths based on demographic, geographic, climatic, traffic, public health, social-distancing-policy adherence, and political characteristics with sufficiently high accuracy. The study reveals that virus pressure is the most important feature in COVID-19 spread for classification and regression analysis. Five other significant features were identified to have the most influence on COVID-19 spread. The combined ensembling approach introduced in this study can help policymakers design prevention and control measures to avoid or minimize public health threats in the future.

## Introduction

Since the World Health Organization (WHO) characterized COVID-19 as a pandemic on March 11, 2020, it has spread to 231 countries and territories, with 698,607,429 coronavirus cases and 6,946,169 deaths by December 3, 2023. The United States is the country most affected by COVID-19, with 109,597,985 confirmed cases and 1,183,777 deaths by the beginning of December 2023 (https://www.worldometers.info/coronavirus/country/us/).

Although no specific treatment or cure for COVID-19 exists, alternatives may reduce the considerable burden on limited healthcare systems and the economic sector. The most promising so far are artificial intelligence techniques like machine learning, data mining, deep learning, and others. Even when vaccines are available, the spread of COVID-19 indispensably relies on some non-medical factors, such as testing, contact tracing, facial coverings, protection of older people, school and workspace closing, public events and other restrictions, etc. Thus, it is necessary and urgent to understand the joint impact of medical, population, weather, and other factors and the restriction policy on COVID-19 spread to guide policymakers in controlling the pandemic.

Since the beginning of the COVID-19 pandemic in early 2020, many machine learning and artificial intelligence techniques have been examined to help fight the disease (e.g.,^1–5^). Though the WHO canceled the pandemic in 2023, COVID-19 still poses a severe risk to the population. Hence, understanding how the disease spreads and the factors affecting its spreading, the number of patients at risk, and the number of death cases is still an important task.

Machine learning (ML) is one of the most advanced artificial intelligence concepts that provides a strategic approach to developing complex algorithmic techniques for advanced data analysis. The ML algorithms can modify their structure based on observed data. There are four main classes of the ML algorithms:

1. Supervised learning. These algorithms use labeled data to predict future events. The learning process starts from a training dataset and develops targeted activity to predict output values.
2. Unsupervised learning. These techniques utilize non-classified or non-labeled datasets. The learning process deduces a function to extract hidden knowledge or a pattern from unlabeled data.
3. Semi-supervised learning. Such algorithms lie between supervised and unsupervised learning techniques, where labeled and non-labeled datasets are used in the training process. Semi-supervised learning can achieve higher accuracy, and these techniques are preferable when a labeled dataset needs appropriate resources for training.
4. Reinforcement learning. These techniques provide feedback to the learning environment to locate errors. They are used to identify the optimal behavior in a given context and increase the performance of the model.

Recently, ML techniques have been widely used to analyze biomedical structured and unstructured data (e.g.,^6–8^). In our previous studies, we studied the effect of the restriction policy on the spread of COVID-19 cases by developing recommendation rules based on the novel ensemble of machine-learning methods such as regression tree and clustering^1^ and introduced an ensemble machine-learning model based on clinical and immunological features for severity risk assessment and post-COVID rehabilitation duration for SARS-CoV-2 patients^2^.

This work uses a combination of supervised and unsupervised ML techniques to develop both classification and regression predictive models for COVID-19 infection and the dataset for confirmed COVID-19 cases and deaths in the USA with a combination of supervised learning algorithms and unsupervised algorithms.

The scientific novelty of the presented work combines contributions in computer science by introducing novel approaches to ensemble models and understanding the risk factors, both medical and non-medical, of COVID-19 spreading, which is essential for making public health decisions that control the pandemic. Thus, we have identified the most significant features having the most influence on COVID-19 spread based on the 46 features presented in the dataset studied. The novelty from the computer science point of view, which is the main focus of the paper, lies in the introduction of novel hybrid hierarchical machine-learning ensembles, which seamlessly integrate both unsupervised and supervised learning approaches for classification and regression analysis. Central to our innovation is the utilization of mathematical expectation to guide the selection of the cut-off coefficient for the stacking ensemble. This dynamic voting mechanism considers the individual scores of weak classifiers within the ensemble, allowing for context-aware decision-making. Rather than relying on a static threshold, our approach computes the average score for each vote, which is then subjected to mathematical expectation to derive an optimal cut-off coefficient. This adaptive strategy ensures that the ensembles of classification are finely tuned to the specific characteristics of the input data, resulting in improved performance across a range of classification tasks.

Furthermore, our research extends beyond the development of classification models themselves to include the training of the cut-off function within the ensemble algorithm. This comprehensive approach not only enhances the accuracy of classification outcomes but also offers insights into the underlying mechanisms driving ensemble decision-making.

Two ensembles are proposed in the paper. In contrast to Ensemble 1, Ensemble 2 trains the cutoff function of the classifier in addition to the trained weak models. The proposed cutting method increases the overall efficiency of the ensemble compared to classical voting, where the class cut-off is done with a constant coefficient of 0.5, thus sharply reducing the efficiency of the algorithm down to approx. 79%. The essence of the algorithm is the selection of the cut-off coefficient. In this case, the voting input contains a vector of independent classifier scores, which, depending on the context, will vote differently. The idea of the method is to determine the average value of the rating at each vote and add it to the list of average ratings. The list of average scores is a set of independent scores. Next, using the mathematical expectation function on this set, the cut-off coefficients are obtained at the output. The obtained cut-off coefficient is close to the optimal class partition coefficient.

The rest of the paper is organized in the following way: Section "Related Works" outlines some recent studies and their analysis, including all the papers that use the dataset examined in this study; Section "Materials and methods" starts with a detailed description of the dataset used and follows with the developed model; Section "Results and discussion" presents the obtained results and related discussion ending with the concluding section.

## Related works

The rapidly evolving disease and the straightforward transmission of virus pathogens have resulted in the development of numerous machine-learning models and applications. S. Solayman et al., in the study^9^, began by precisely preparing knowledge obtained from the Israeli Ministry of Health open-source website for classifiers. Experiments demonstrated that the hybrid convolutional neural network and long short-term memory algorithm with the SMOTE approach achieved the best results for classifying the introduced data. Satisfactory outcomes led to implementing an application to forecast COVID-19 infections for users, providing feedback based on entered symptoms. Another application of machine learning in the fight against COVID-19 is highlighted in the paper^3^, where the authors predict the condition of coronavirus-infected patients. Experiments utilized two datasets: demographic and clinical data of patients (n = 11,712) and demographic data, clinical information, and patient blood test results (n = 602) to develop predictive models and identify key features. Subsequently, the performance of eight different machine learning algorithms was compared. The research used demographic, clinical, and blood data. Experiments demonstrated that C-reactive protein, lymphocyte ratio, lactic acid, and serum calcium significantly influence the prognostic predictions of COVID-19. A study conducted in South Korea^10^ involving 10,237 patients revealed that factors like age over 70, moderate or severe disability, comorbidities, and male gender are strongly associated with an increased risk of mortality from COVID-19. Through machine learning analysis, Lasso and Linear Support Vector Machine (SVM) models exhibited higher sensitivity and specificity in predicting mortality. The developed predictive model can classify patients rapidly under limited medical resources during a pandemic.

One consistent observation from the ongoing research on COVID-19 data is the variability in the application of classification methods across different countries. Experiments in the study^11^ reveal that the Prophet model demonstrated sufficient accuracy in predicting cases in the USA while considering Brazil or India; the Autoregressive Integrated Moving Average model performed better. The superiority of a deep learning model, including the Neural Prophet model, is confirmed by the study^12^ conducted in 2022. Another research^13^ illustrates the differences in applied statistical models depending on the location. The article examined multilayer perceptron, vector autoregression, and linear regression to predict the epidemic caused by the SARS-CoV-2 virus, utilizing data from Asian countries obtained from the Johns Hopkins University data repository. Drawing on data from Mexico, Muhammad et al.^14^ developed supervised machine-learning models for COVID-19 infection using various classification models, examining correlations between input features beforehand. According to the research, the highest accuracy is associated with decision trees at 94.99%, the highest sensitivity (93.34%) with the Support Vector Machine model, and Naive Bayes exhibits the highest specificity at 94.30%.

A lack of accurate data on COVID-19 hinders the standard techniques for predicting the consequences of an epidemic. Considering this knowledge, Tiwari et al.^15^ applied meta-analysis based on artificial intelligence, utilizing machine learning algorithms such as Naive Bayes, SVM, and Linear Regression to predict the trends of the global epidemic caused by the SARS-CoV-2 virus. Among the discussed techniques, Naive Bayes yielded the most satisfying results, demonstrating high effectiveness in predicting future values with less mean absolute error and mean squared error. A comprehensive study employing diverse artificial intelligence strategies is described in reference^16^, where long short-term memory, multilayer perceptron, adaptive neuro-fuzzy inference system, and recurrent neural network were employed. The analysis of the effectiveness of the considered methods focuses on results obtained from calculating mean squared error (RMSE), mean absolute percentage error (MAPE), mean absolute error (MAE), and R^2^ coefficient of determination (R^2^). The results indicate that for Bidirectional long short-term memory (LSTM) and artificial neural network models, R^2^ values range from 0.64 to 1. Autoregressive Integrated Moving Average (ARIMA)and LSTM models demonstrated the highest MAPE errors. Another approach is characterized by the work of S. A.-F. Sayed et al.^17^, who built a model predicting various levels of severity risk for COVID-19 using the analysis of chest X-ray images. Deeply trained CheXNet model and hybrid feature extraction techniques were applied in experiments. The study showed that the XGBoost classifier performed best with combined features (PCA + RFE), generating 97% accuracy, 98% precision, 95% recall, 95% F1-score, and 100% ROC-AUC. In the study, SVM demonstrated results that were equally satisfying as those of XGBoost. In the paper^18^, Atta-ur-Rahman et al. directed their attention towards a mathematical model based on a cloud-based smart detection algorithm using a support vector machine. The obtained solutions oscillated around 98.4% accuracy with a 15-fold cross-validation. The comparison conducted in the study suggests that the proposed model exhibits greater accuracy and efficiency.

In one of the reviews encompassing 160 studies^19^, a compilation of machine learning techniques from various sources such as Springer, IEEE Xplore, and MedRxiv was made. Two categories of machine learning were outlined: deep learning and supervised learning. Statistics indicate that deep learning is employed in 79% of cases, with 65% utilizing convolutional neural networks (CNN) and 17% using Specialized CNN. Focusing on supervised learning, only 16% of analyses were observed, predominantly using Random Forest, Support Vector Machine, and regression algorithms. On the other hand, studies from 2021 by Kwekha-Rashid et al.^5^ demonstrated that better learning results could be observed using supervised learning, characterized by high accuracy at 93%. A comparison of research results^4^ on machine learning applications in the context of COVID-19 revealed that recurrent neural networks, deep diagnostic models, various contact tracing, medical diagnostics, and drug development-related algorithms were effective. Forecasting models achieved high correlations and diagnostic models analyzing computer tomography and X-ray images demonstrated accuracy at 99%. The authors emphasized that limitations related to the lack of full access to patient data and algorithm imperfections highlight the need for the involvement of government agencies in facilitating the acquisition of COVID-19-related data. Alballa and Al-Turaiki^20^ focused on COVID-19 diagnosis and predicting severity and mortality risk using machine learning algorithms. The authors note that most machine learning algorithms are supervised learning models, which are more straightforward and more understandable. The referenced article states the need for further research, especially in identifying optimal screening models for COVID-19 and creating a comparative dataset. The limitation is the use of unbalanced datasets, requiring effective techniques to deal with this issue, and the potential integration of different types of data, necessitating further research for precise COVID-19 prediction.

Tkachenko et al.^6^ aimed to increase the performance of prediction tasks using combined RBF-SGTM neural-like structures. They developed a committee of non-iterative artificial intelligence tools for regression analysis. Using the developed committee for insurance cost prediction allows the authors to decrease training and test errors and increase accuracy with a slight increase in the training procedure time. The authors conclude that the developed neural-like structures can be used to solve regression and classification tasks with large volumes of data for different application areas.

This research uses publicly available COVID-19 outbreaks and potential predictive features in the USA dataset^21^ (see section "Dataset description"). So far, six papers^22–27^ have reported using this dataset in their studies. Two of them^22,23^ studied a vital problem of missing and incomplete data for the medical domain. In this domain, the accuracy of the data is critical, while missing values are a typical occurrence there for various reasons. Pathak et al.^22^ have studied various techniques for missing data imputation using COVID-19 data from^21^. They evaluated the performance of four mostly adopted data imputation techniques, viz., multivariate imputation by chained equations, expectation and maximization algorithm, mean, and KNN. The authors conclude that KNN is an imputation approach that is expected to be a good fit for dealing with missing data in the healthcare industry. Batra et al.^23^ developed an ensemble strategy for missing values imputation in health data. The authors introduced an ensemble imputation model comprising simple mean imputation, KNN imputation, and iterative imputation methods. The proposed ensemble combines the mentioned methods to opt for the ideal imputation strategy based on attribute correlations on missing value features. S. Batra et al. introduced the Ensemble Strategy for Missing Values to identify unbiased and accurate statistical modeling predictions. They used the performance metrics generated using the eXtreme gradient boosting (XGB) regressor, random forest regressor, and support vector regressor. The authors concluded that the proposed ensemble strategy is the most suitable option for imputing missing values. When passed to the XGB regressor for performance evaluation, the imputed dataset has the mean absolute error values of 60.81, 54.06, and 49.38 for 5000, 10,000 and 20,000 records datasets.

Paper^24^ identifies direct causes using the intervention target variable. As a target variable, the authors used the number of COVID cases from the mentioned COVID dataset^21^. Du et al.^24^ developed the invariant prediction framework using the invariance principle based on linear models. They used the conditional distribution of the target variable, given its causal parents are invariant across multiple environments or experimental conditions. The developed approach outperformed various baselines for predicting COVID cases in 8 areas, mainly from the US East Coast.

Gholamalian et al.^25^ studied the problem of determining the infection status of individuals using sparse group-level tests by extending graph-coupled hidden Markov models with infection statuses of the individuals as the hidden states and the group test results as the observations. The developed model^25^ was separately tested daily to predict the status after 15 days of the beginning of the spread. The AUC metrics value was 0.98 on day 16 and remained above 0.80 until day 128.

The forecasting of the COVID-19 dynamics under imperfect vaccination was studied by Wang et al.^26^ by combining a mechanistic ordinary differential equation model and a generalized boosting machine learning model. The ordinary differential equation model was utilized for infectious class prediction. In contrast, the machine-learning model was used to predict how public health policies and mobility data affect the transmission rate in the former model, including the post-vaccination period. The authors reached the average mean absolute percentage error value of 14.88% when considering human mobility data for predicting the number of daily infected cases up to 35 days in the future.

Du and Xiang^27^ aimed to identify different forms of invariance to facilitate prediction in unseen environments. They used linear structural causal models and introduced an invariant matching property, an explicit relation, to capture interventions through an additional feature. Such an alternative form of invariance makes it possible to develop a unified treatment of general interventions on the response and the predictors. The authors utilized the dataset^21^ to predict the number of COVID cases using the 12 temporal features for the time interval from March 1, 2020, to September 30, 2020.

## Materials and methods

### Dataset description

This study used publicly available COVID-19 outbreaks and potential predictive features in the USA dataset^21^. The dataset provides information related to the outbreak of COVID-19 disease in the United States, including data from each of 3142 US counties from the beginning of the outbreak (January 2020) until June 2021. This data was collected from many public online databases and includes the daily number of COVID-19 confirmed cases and deaths, as well as 46 features that may be relevant to the pandemic dynamics: demographic, geographic, climatic, traffic, public-health, social-distancing-policy adherence, and political characteristics of each county^21^. The dataset contains the number of confirmed COVID-19 cases and deaths and 46 factors that may be relevant to the pandemic dynamics in each county and for each day since the beginning of the outbreak (Table 1). Haratian et al.^21^ also prepared a processed version of the dataset, where the missing values are imputed and the abnormal values, e.g., negative counting values, are fixed. The detailed description of the dataset and the data origin and processing are described in^21^. The target variables are the COVID-19 confirmed cases and deaths numerical values. The 46 features can be divided into the following classes:

- Fixed features
  o Demographic features.
  o Health facilities and risk factors features.
  o Geographic features.
  o Economic and other features.
- Temporal features
  o Climate features.
  o Social distancing features.
  o Other features: daily state tests, weekly admission, weekly reported total inpatient beds, weekly occupied inpatient beds, weekly reported total ICU beds, weekly occupied ICU beds, the percentage of vaccinated residents, and the virus pressure.

**Table 1:** Description of the features^21^.

#	Variable name	Description	Percentage of values available in the dataset	Type	Finest spatial scale	Date of access to the data source
Target variables						
(1)	COVID-19 confirmed cases	Number of daily COVID-19 confirmed cases	100	Real	County	Jun 10, 2021
(2)	COVID-19 deaths	Number of daily COVID-19 deaths	100	Real	County	Jun 10, 2021
Fixed features						
(3)	Total population	Total population	100	Real	County	Apr 17, 2020
(4)	Population density	Population per square mile	100	Real	County	–
(5)	Proportion female	Total number of females divided by the total population	100	Real	County	–
(6)	Age distribution	Percentage of residents in the age groups: 0–4, 5–9, 10–14, 15–19, 20–24, 25–29, 30–34, 35–39, 40–44, 45–49, 50–54, 55–59, 60–64, 65–69, 70–74, 75–79, 80–84, 85 and older	100	Real vector (18 values, that add up to 1)	County	Apr 17, 2020
(7)	Education level distribution	Percentage of residents with different levels of education: 'less than high school diploma', 'high school diploma', 'some college or associate's degree'	100	Real vector (4 values, that sum to 1)	County	Aug 18, 2020
(8)	Median household income	–	100	Real	County	May 4,2020
(9)	GDP per capita	Gross Domestic Product per capita (economic output divided by the population)	100	Real	County	Apr 27,2020
(10)	Area	Area in square miles	100	Real	County	May 6,2020
(11)	Latitude	Latitude of the county barycenter	100	Real	County	May 1,2020
(12)	Longitude	Longitude of the county barycenter	100	Real	County	May 1,2020
(13)	Housing density	Number of housing units per square mile (Including houses, apartments/flats, mobile homes, and other housing units)	100	Real	County	Apr 17,2020
(14)	Academic population ratio	Total number of residents who are currently university and college students or staff, divided by the total population	100	Real	County	May 4,2020
(15)	Immigrant students ratio	Total number of students who study in this county but are residents of the other states, divided by the total county population	100	Real	County	Sep 10,2020
(16)	Hospital bed ratio	Number of Hospital beds divided by the total population	100	Real	County	May 11,2020
(17)	Intensive care unit (ICU) bed ratio	Number of ICU beds divided by the total population	98	Real	County	May 11,2020
(18)	Ventilator capacity ratio	Number of ventilators divided by the total population	98	Real	County	May 11,2020
(19)	Percent of smokers	Percentage of adult smokers	100	Real	County	May 11,2020
(20)	Percent of diabetes	Percentage of diabetic adults	100	Real	County	May 11,2020
(21)	Religious congregation ratio	Number of active members of Religious congregations divided by the total population	99	Real	County	Apr 17,2020
(22)	Number of meat plants	Number of meat processing plants	100	Discrete	County	Aug 20,2020
(23)	Airport distance	Distance to the nearest international airport with average daily passenger load more than ten	100	Real	County	May 1,2020
(24)	Passenger load ratio	Average daily passenger load of that nearest international airport divided by the total population	100	Real	County	May 20,2020
(25)	Percent of insured residents	Percentage of health insured residents	99	Real	County	May 11,2020
(26)	Death ratio	Number of deaths divided by the total population	97	Real	County	June 21,2020
(27)	Political party	The political party of the governor of each state (0 for Republican and 1 for Democratic)	100	Discrete	State	Apr 17, 2020
(28)	Population ratio in state	Total population of the county, divided by its state population	100	Real	County	-
Temporal features						
(29)	Precipitation	Daily precipitation	73	Real	County	June 10, 2021
(30)	Temperature	Daily average temperature	59	Real	County	June 10, 2021
(31)	Daily state test	Number of total COVID-19 tests performed at each day in the state of the county (including antibody, antigen, and PCR tests)	91	Integer	State	June 10, 2021
(32)	Percent of vaccinated residents	Percent of residents who are fully vaccinated (have second dose of a two-dose vaccine or one dose of a single-dose vaccine)	99	Integer	County	June 10, 2021
(33)	Weekly admission	Weekly average number of adult or pediatric patients who were admitted to an inpatient bed in the county who had confirmed COVID-19 at the time of admission	31	Real	County	June 10, 2021
(34)	weekly reported total ICU beds	Weekly average number of total number of staffed inpatient ICU beds reported by the hospitals in the county	46	Real	County	June 10, 2021
(35)	weekly occupied ICU beds	Weekly average number of total number of staffed inpatient ICU beds that are occupied, reported by the hospitals in the county	45	Real	County	June 10, 2021
(36)	weekly reported total inpatient beds	Weekly average number of total number of staffed inpatient beds (including ICU beds) reported by the hospitals in the county	46	Real	County	June 10, 2021
(37)	weekly occupied inpatient beds	Weekly average number of total number of staffed inpatient beds that are occupied, reported by the hospitals in the county	46	Real	County	June 10, 2021
(38)	Social distancing travel distance grade	Percent change in average distance traveled compared to pre-COVID-19-period (range from A to F) A: > 70% decrease B: 55–70% decrease C: 40–55% decrease D: 25–40% decrease F: < 25% decrease or increase	99	Nominal	County	June 10, 2021
(39)	Social distancing visitation grade	Percent change in non-essential visitation compared to pre-COVID-19 period (range from A to F) A: > 70% decrease B: 65–70% decrease C: 60–65% decrease D: 55–60% decrease F: < 55% decrease or increase	82	Nominal	County	June 10, 2021
(40)	Social distancing encounters grade	Percent change in human encounters compared to pre-COVID-19 period (range from A to F) A: > 94% decrease B: 82%-94% decrease C: 74%-82% decrease D: 40%-74% decrease F: < 40% decrease or increase	99	Nominal	County	June 10, 2021
(41)	Social distancing total grade	Average numerical score of the previous three social distancing factors	99	Nominal	County	June 10, 2021
(42)	Retail and recreation mobility percent change	Percent change in mobility trends in retail shops and recreation centers (including places like restaurants, shopping centers, museums, and libraries) compared to pre-COVID-19 period	49	Real	County	June 10, 2021
(43)	Grocery and pharmacy mobility percent change	Percent change in mobility trends in grocery stores and pharmacies (including places like grocery markets, food warehouses, farmers markets, specialty food shops, drug stores, and pharmacies) compared to pre-COVID-19 period	44	Real	County	June 10, 2021
(44)	Parks mobility percent change	Percent change in mobility trends in parks (including local and national parks, public beaches, marinas, dog parks, plazas, and public gardens) compared to pre-COVID-19 period	18	Real	County	June 10, 2021
(45)	Transit stations mobility percent change	Percent change in mobility trends in transit stations (representing public transport hubs like taxi stands, bus, train, and subway stations) compared to pre-COVID-19 period	28	Real	County	June 10, 2021
(46)	Workplaces mobility percent change	Percent change in mobility trends in places of work compared to pre-COVID-19 period	74	Real	County	June 10, 2021
(47)	Residential mobility percent change	Percent change in mobility trends in places of residence compared to pre-COVID-19 period	42	Real	County	June 10, 2021
(48)	Virus pressure	A measure for virus transmission from neighboring counties, defined as the weighted average of the number of confirmed cases in the adjacent counties (i.e., that share a border with this county)	100	Real	County	–

The details of the dataset are listed in Table 1.

### Proposed supervised-unsupervised ensemble model

We used R version 4.1.2 to conduct this research, run on Spark 3.0.0, 8 cores.

Since the dataset contains 46 fixed and temporal features, it could be challenging to build a good predicting ML model or even to select the most important features of a different nature. Since these data are of a different nature (demographic, geographic, economic, climate, social etc., see Section "Dataset description"), we developed a naturally inspired multimodal-like ML model that combines both supervised and unsupervised learning. Like the human brain combines input signals of different origins, e.g., audial and visual, in the temporal lobe, our ensemble combines inputs from different feature clusters in a hybrid classifier. The working hypothesis is that it is insufficient to select important features, but we should combine them into clusters of similar impact on the COVID-19 spread. Next, these clustered features provide an aggregated input to an ensemble classifier to increase the prediction accuracy and resilience.

The following performance metrics were used to evaluate the model performance: accuracy, F1-score, the area under the receiver operating characteristic curve (ROC-AUC) for the classification task, and mean squared error (MSE), ROC-AUC, Model Performance Predictor (MPP) for the regression task. It tracks the predictive performance metric of the model.

In the first step of the research, data preprocessing was done. This included one-hot encoding for two features, missing data removal and feature selection. Missing data imputation is not implemented; only missing data removal was used based on the low level of missing data (see Table 1). The imputed using the KNN imputer dataset is also available in^21^, and the potential usage of this dataset will be discussed later in this paper. As a result, 69 features were obtained. Primary Component Analysis (PCA) was used to reduce the dimensionality. PCA results in the 60 primary components that do not substantially affect the dimensionality. This finding confirms the research hypothesis on the necessity of combining unsupervised and supervised learning to reduce the dimensionality of the input data and potentially increase the accuracy and robustness of the prediction model.

The next step was to use a complex ensemble for the classification task with three labels: min risk for confirmed cases, mid risk and huge risk. Two hybrid stacking ensembles are proposed.

#### Stacking supervised-unsupervised ensemble

**Figure 1:**
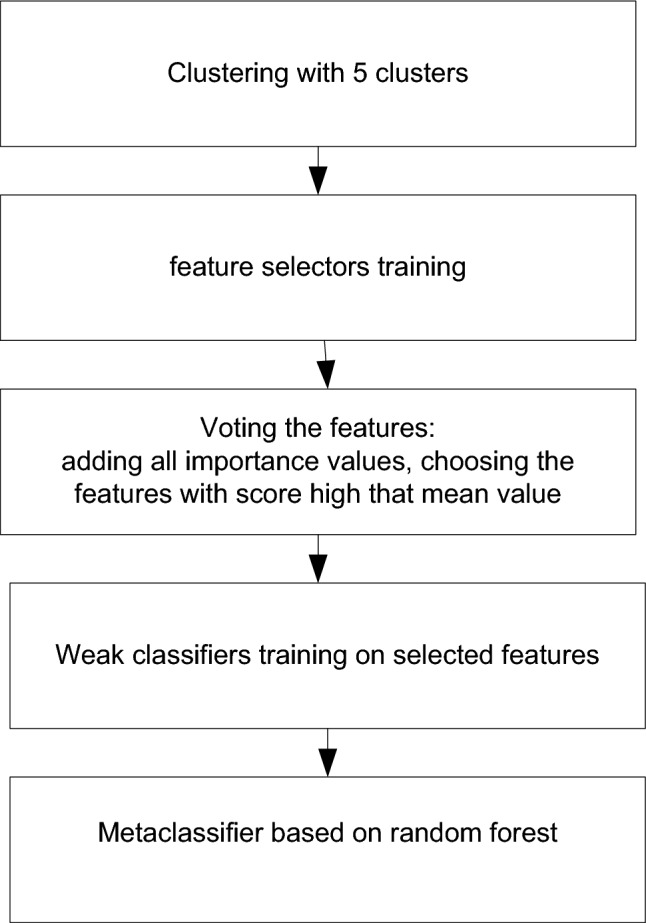
The proposed hierarchical hybrid stacking classifier (Ensemble 1).

The first ensemble is built on the classical stacking approach when only class probabilities and the corresponding target values are fed to a meta-classifier. A hierarchical hybrid classifier was developed (Fig. 1), which includes the following three levels:

1. Clustering of the input data using the k-means algorithm.
2. Selecting the most important features in each obtained cluster using Boruta, decision tree and Random forest.
3. Building a stacking ensemble using the selected features for each cluster using the Random Forest algorithm as a meta-model. Logistic regression, KNN, SVM with linear kernel, naïve Bayes, decision tree and SVM with RBF kernel were used as weak classifiers.

First, one-hot encoding was implemented for categorical features such as age, education level, country, state, etc. The elbow method was used to select the appropriate number of clusters. Fourteen features were selected after voting for Boruta, random forest, and decision tree feature selectors.

The decision tree returns the feature weight as the criterion for evaluating features. It allows building a ranked list of selected features using different measures. In our case, CART was used for feature selection, with the Gini index as a measure.

Random Forest is an ensemble of numerous training-sensitive algorithms (decision trees). These algorithms have a slight offset. The bias of the training method is the deviation of the average response of the trained algorithm from the response of the ideal algorithm. Each of these classifiers is built on a random subset of objects and a random subset of features.

Boruta is a heuristic algorithm for selecting significant features based on the use of Random Forest. At each iteration, those features are removed for which the Z-measure is less than the maximum Z-measure among the added features. To get the Z-measure of a feature, it is necessary to calculate its importance, obtained using the built-in algorithm in Random Forest, and divide it by the standard deviation of the feature importance. Added features are obtained as follows: the characteristics available in the selection are copied, and then each new attribute is filled by shuffling its values. This procedure is repeated several times to get statistically significant results, and variables are generated independently at each iteration.

Next, the Jaccard index is used for feature selector voting.

Next, voting for the features is developed. First, all important values are added. Next, the features with scores higher than the mean value are chosen. After that, 15 diverse classifiers were used, and 9 of the strongest were selected.

#### Modified stacking supervised-unsupervised ensemble

**Figure 2:**
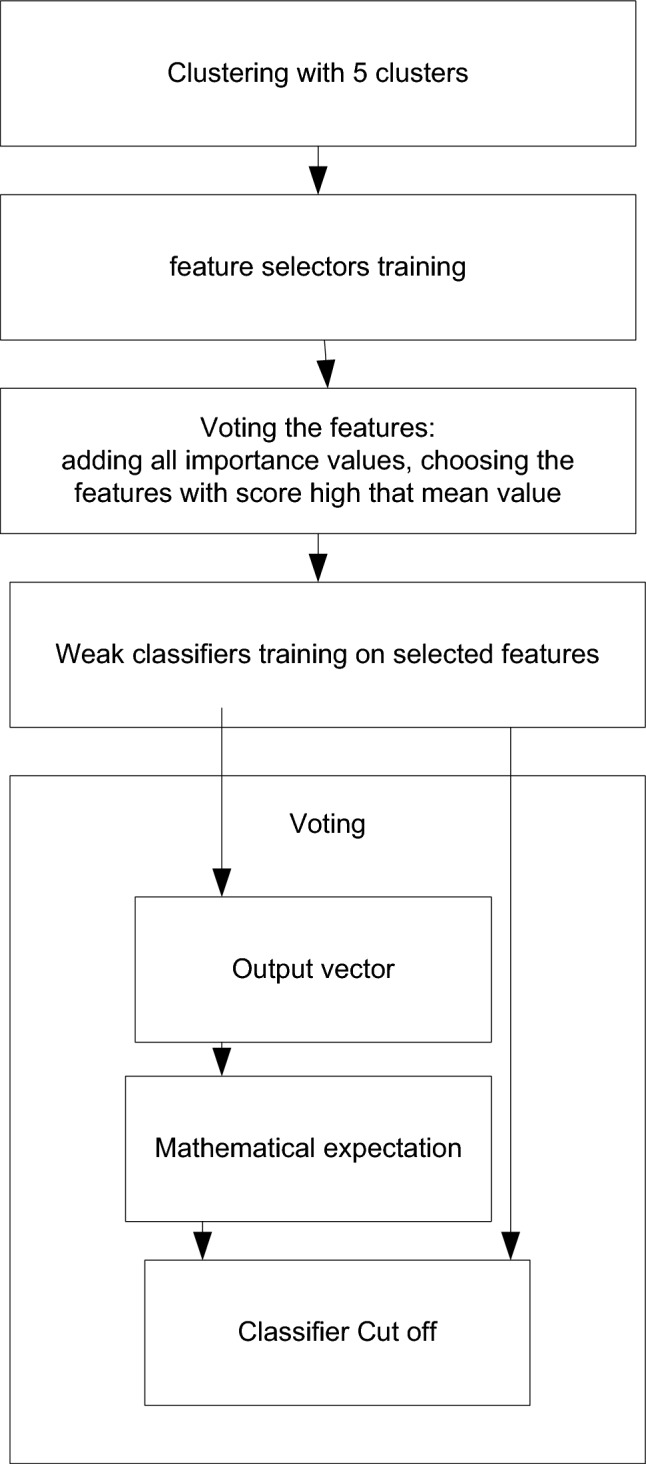
Modified hierarchical hybrid stacking classifier (Ensemble 2).

The second ensemble utilizes a modified stacking approach when all datasets and transformed outputs of the weak classifiers are fed to a meta-classifier. Figure 2 depicts the structure of the proposed ensemble and the data transformation.

In contrast to Ensemble 1, Ensemble 2 trains the cutoff function of the classifier in addition to the trained weak models. The proposed cutting method increases the overall efficiency of the ensemble compared to classical voting, where the class cut-off is done with a constant coefficient of 0.5, thus sharply reducing the efficiency of the algorithm to approx. 79%. The essence of the algorithm is the selection of the cut-off coefficient. In this case, the voting input contains a vector of independent classifier scores, which will vote differently depending on the context. The idea of the method is to determine the average value of the rating at each vote and add it to the list of average ratings. The list of average scores is a set of independent scores. Next, the cut-off coefficients are obtained at the output using the mathematical expectation function on this set. The obtained cut-off coefficient is close to the optimal class partition coefficient.

For each classifier and regressor, fivefold nested cross-validation was used. Each fold is constituted by two arrays: the first one is related to the training set, and the second one is related to the test set.

**Figure 3:**
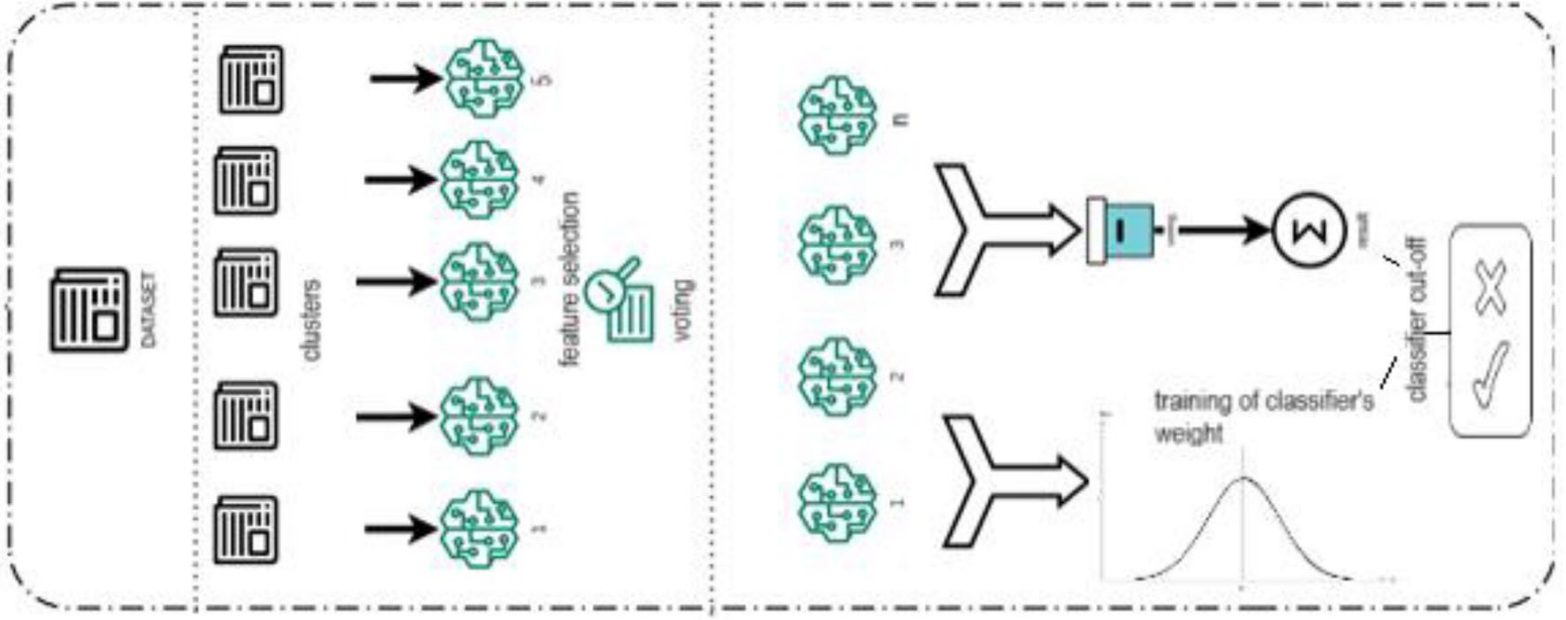
The general pipeline.

The general pipeline is given in Fig. 3.

## Results and discussion

### Classification task

The target classes for this task were three classes with a risk of new COVID cases. Nine single classifiers, viz., Logistic Regression (GM), Decision Tree, SVM with linear kernel, k-nearest neighbors (KNN), eXtreme Gradient Boosting (XGBoost), SVM with Radial kernel (RBF), Random Forest, Naïve Bayes, and Multilayered perceptron with three hidden layers and four neurons inside of each layer (Ml (c(4, 3, 3)), were used to compare the performance of the proposed ensembles.

Table 2 lists the most important features for the new COVID-19 case classification according to Boruta, Random Forest, and Decision Tree feature selectors (for each feature description, see Table 1). The listed features can help decision-makers select factors affecting COVID-19 spread and thus optimize medical care and/or restriction policy to minimize the epidemic impact, considering all aspects of human well-being.

**Table 2:** Important classification features after voting Boruta, Random Forest and Decision Tree feature selectors.

Feature	Importance
Virus pressure	0.7093872
Total population	0.1176112
Country fips	0.1094524
Percentage of residents in the age group 25–29	0.0126625
Temperature	0.0124976
Social distancing encounters grade	0.0099895
Immigrant students ratio	0.0068993
Airport distance	0.0063710
Housing density	0.0040341
Daily state test	0.0033927
Longitude	0.0032784
Intensive care unit (ICU) bed ratio	0.0031758
Population ratio in state	0.0011955
Workplaces mobility percent change	0.0000528

The classification performance metrics for 9 weak classifiers and the proposed ensembles are summarized in Table 3. As one can see from the table, the best classification results were obtained in the case of the KNN model, with Accuracy = 0.816, ROC-AUC = 0.797, and F1-score = 0.814. Using the developed ensembles allows us to increase all the metrics substantially. Thus, in the case of Ensemble 1, Accuracy was raised to 0.895, ROC-AUC to 0.897, and F1-score to 0.897. The proposed cut-off voting improvement in Ensemble 2 further increased all the metrics compared to Ensemble 1 by approx. 2% (Accuracy, ROC-AUC, and F1-score values are 0.912, 0.916, and 0.916 correspondingly). Hence, the developed hybrid hierarchical classifiers outperform single classification algorithms by more than 10% and are well-suited for COVID-19 spread prediction in real life.

**Table 3:** Classification performance of the weak and ensemble classifiers.

Model	Accuracy	ROC-AUC	F1-score
GM	0.614	0.616	0.616
Decision tree	0.713	0.715	0.715
SVM linear	0.733	0.732	0.735
KNN + 10-folds cross-validation	0.816	0.797	0.814
XGBoost	0.797	0.797	0.806
RBF	0.675	0.719	0.716
Random forest	0.725	0.728	0.728
Naïve Bayes	0.721	Nfble0.728	0.724
Ml (c(4, 3, 3))	0.733	0.732	0.735
Ensemble 1	**0.895**	**0.897**	**0.897**
Ensemble 2	**0.912**	**0.916**	**0.916**

Significant values are given in bold.

Dynamic voting based on mathematical expectation is used. In addition to the trained models themselves, the cutoff function of the classifier is trained in this algorithm. The traditional stacking is based on averaging indicators, and there is a cut-off by class with a constant coefficient of 0.5; then, the efficiency of the algorithm drops sharply to ~ 79%. The proposed cutting method increases the overall efficiency of the ensemble by several percent. The essence of the algorithm is to choose a cut-off coefficient. In the case of this work, the voting input contains a vector of independent classifier scores, which will vote differently depending on the context. The idea of the method is to calculate the average score for each vote and add it to the list of average scores. The list of average grades is a set of independent grades on which the mathematical expectation function is applied. We got a cut-off coefficient close to the optimal class separation coefficient at the output.

We used the nested fivefold cross-validation technique to perform additional tests, as described in^28^. Nested cross-validation was used to validate the findings obtained using the proposed approach in addition to the usual fivefold cross-validation. Though this approach has its limitations, e.g., the assumption of the data split independence, it is widely used across the ML community. The difference between the Accuracy values across the five folds was 0.018. Next, we performed a more robust statistical test, viz. Kolmogorov–Smirnov normality test. The obtained p-value was 0.793.

Table 4 shows the efficiency of proposed ensembles for the whole dataset and for selected features. Selecting features allows for increasing the total analyzed metrics.

**Table 4:** Classification performance of the whole dataset and selected features.

Model	For the whole dataset			For selected features		
	Accuracy	ROC-AUC	F1-score	Accuracy	ROC-AUC	F1-score
Ensemble 1	0.884	0.881	0.885	0.895	0.897	0.897
Ensemble 2	0.882	0.884	0.883	0.912	0.916	0.916

### Regression task

For the regression task, the following regression models were used: linear model, polynomial regression, regression tree with CART algorithm, Gradient boosted tree, random forest, l1 regularization for the linear model, and l2 regularization for the linear model. These models aimed to predict the number of confirmed COVID-19 cases and deaths. Table 5 summarizes the most important features affecting the prediction of the COVID-19 spread.

**Table 5:** Important features for the regression task after voting Boruta, Random Forest and Decision Tree feature selectors.

Feature	Importance
Virus pressure	0.3774971
Social distancing total grade	0.2506208
Total population	0.1412967
Area	0.1068608
Retail and recreation mobility percent change	0.0627281
Number of meat plants	0.0159237
Percent of insured residents	0.0129029
Airport distance	0.0099794
Median household income	0.0072303
Longitude	0.0050858
Daily state test	0.0045425
Temperature	0.0043822
Population ratio in state	0.0009497

As it follows from the comparison of Tables 2 and 5, virus pressure, i.e., a measure for virus transmission from neighboring counties, defined as the weighted average of the number of confirmed cases in the adjacent counties, is the most important feature for classification and regression analysis. Besides, there is a subset of common features, which were recognized as the most important in these two studies, viz., (i) the total population of the county—the second most important common feature, (ii) distance to the nearest international airport with average daily passenger load more than ten, (iii) daily average temperature, (iv) the longitude of the county barycenter, (v) number of total COVID-19 tests performed at each day in the state of the county, and (vi) population ratio in the state. As we can see, the COVID-19 spread is affected by various factors: epidemiological, like the virus pressure; demographic, like the total population and population density; social, like the distance to the nearest international airport; climate, like daily average temperature; geographical, like the longitude of the county barycenter, and medical like the number of total COVID-19 tests performed at each day. These findings can help epidemiologists to analyze the spread and lifecycle of the virus and decision makers to select the most important restriction factors and limitations to prevent the spread of the disease.

Other factors affecting the number of COVID-19 cases and deaths—as seen in Table 4—are mainly social features, like social distancing, percentage of health-insured residents, median household income, and percent change in mobility trends in retail shops and recreation centers. The analysis of Table 2 reveals that while speaking on the classification, there are some additional factors affecting the chance of getting infected with coronavirus, viz., percentage of residents in the age group 25–29, immigrant student ratio, intensive care unit bed ratio, and the percent change in human encounters compared to pre-COVID-19 period.

Table 6 lists the regression task performance evaluation for the six most common regression models and the proposed ensemble.

**Table 6:** Regression task performance metrics for weak and ensemble classifiers.

Model	MSE	ROC-AUC	MPP
Lm linear model	133.0	0.455	28.4
regression tree	131.3	0.469	22.7
Grad boosted tree	112.6	0.609	18.8
Random forest	126.4	0.507	17.73
L1 lm	121.3	0.491	23.6
L2 lm	125.3	0.509	17.7
Ensemble 1	**101.3**	**0.790**	**13.1**
Ensemble 2	**101.1**	**0.795**	**12.9**

Significant values are given in bold.

The proposed hybrid hierarchical ensemble combining both supervised and unsupervised learning allows us to increase the accuracy of the regression task by 11% in terms of MSE, 29% in terms of the area under the ROC, and 43% in terms of the MPP metric. Indeed, the ROC-AUC value increased from 0.609 for the best traditional regression model (Gradient Boosted Tree) up to 0.790 in the case of the proposed Ensemble; MSE decreased from 112.6 down to 101.3, and MPP from 18.8 to 13.1 respectively. Thus, using the proposed approach, it is possible to predict the number of COVID-19 cases and deaths based on demographic, geographic, climatic, traffic, public health, social-distancing-policy adherence, and political characteristics with sufficiently high accuracy.

Besides, we used a nested fivefold cross-validation technique^28^ to perform a grid search hyperparameters optimization. The tuning parameter α was set to a constant value of 1. RMSE was used to select the optimal model using the smallest value. The final values used for the model were α = 1 and λ = 0.211 with the MAE metrics of 9.51, RMSE of 20.11 and R^2^ value of 0.76.

The developed way of cutting off the classifier or regressor, which is the part of the ensemble, increases the overall efficiency of the ensemble by several percent. A vector of models with different contextual characteristics can provide reasonable generalized estimates.

Table 7 shows the efficiency of proposed ensembles for the whole dataset and for selected features. Feature selection allows for increasing all the analyzed metrics.

**Table 7:** Regression performance of the whole dataset and selected features.

Model	For the whole dataset			For selected features		
	MSE	ROC-AUC	MPP	MSE	ROC-AUC	MPP
Ensemble 1	111.2	0.509	17.73	101.3	0.790	13.1
Ensemble 2	109.8	0.607	16.6	101.1	0.795	12.9

## Conclusions

This paper introduces two hybrid hierarchical machine-learning ensembles, which combine supervised and unsupervised learning algorithms for classification and regression predictions of the COVID-19 spread. The developed ensembles are based on a combination of supervised learning algorithms and unsupervised algorithms with a new method of selecting the cut-off coefficient based on the mathematical expectation of the weak classifier predictors. The study utilizes publicly available COVID-19 outbreak and potential predictive features in the USA dataset, which provides daily information related to the outbreak of COVID-19 disease in the US, including data from each of 3142 US counties from the beginning of the epidemic (January 22, 2020) until June 10, 2021.

The developed hybrid hierarchical classifiers outperform single classification algorithms by more than 10% and are well-suited for COVID-19 spread prediction in real life. In the case of Ensemble 1, the achieved Accuracy metric was 0.895, ROC-AUC—0.897, and F1-score—0.897. The proposed cut-off voting improvement in Ensemble 2 further increased all the metrics compared to Ensemble 1 (Accuracy, ROC-AUC, and F1-score values are 0.912, 0.916, and 0.916, respectively).

Central to our innovation is using mathematical expectation to guide the selection of the cut-off coefficient in Ensemble 2. This dynamic voting mechanism considers the individual scores of weak classifiers within the ensemble, allowing context-aware decision-making. Rather than relying on a static threshold, our approach computes the average score for each vote, which is then subjected to mathematical expectation to derive an optimal cut-off coefficient. This adaptive strategy ensures that the ensembles of classification are finely tuned to the specific characteristics of the input data, resulting in improved performance across a range of classification tasks.

The proposed hybrid hierarchical ensemble combining both supervised and unsupervised learning allows us to increase the accuracy of the regression task by 11% in terms of MSE, 29% in terms of the area under the ROC, and 43% in terms of the MPP metric. The ROC-AUC value increased from 0.609 to 0.790; MSE decreased from 112.6 to 101.3, and MPP from 18.8 to 13.1, respectively. Thus, using the proposed approach, it is possible to predict the number of COVID-19 cases and deaths based on demographic, geographic, climatic, traffic, public health, social-distancing-policy adherence, and political characteristics with sufficiently high accuracy.

The model described in^26^ was able to predict the number of daily infected cases up to 35 days in the future, with an average mean absolute percentage error of 20.15% with further improvement to 14.88% if combined with human mobility data. In our study, we used the MSE metric instead, so the results cannot be compared directly. MAE value obtained during nested cross-validation is 9.51. The obtained AUC value for this research is 0.916 for the classification task and 0.795 for the case of regression analysis. A similar AUC value (0.80) was also reported by Zahra Gholamalian et al. to predict the statuses over time, viz. for the classification, in^25^.

Wang et al.^26^ determined the policies of restrictions on gatherings, testing and school closing as the most influential predictor variables. In this paper, the most influential predictor variables are virus pressure, social distancing total grade, total population, area, and retail and recreation mobility percent change. Virus pressure was also reported as the key indicator for the number of COVID-19 cases in each county^24^.

The study shows that the most important feature in COVID-19 spread is virus pressure for classification and regression analysis. Besides, there is a subset of common features which were recognized as the most important in these two studies:

- the total population of the county—the second most important common feature,
- distance to the nearest international airport,
- daily average temperature,
- the longitude of the county barycenter,
- number of total COVID-19 tests performed each day in the state of the county,
- population ratio in the state.

These findings can help practitioners analyze the spread and lifecycle of the virus, and decision-makers select the most critical restriction factors and limitations to prevent the spread of the disease. COVID-19 model predictions play a crucial role in shaping public health practices and informing policy decisions, offering insights into the potential trajectory of the pandemic and the effectiveness of various interventions. Models can help predict the demand for healthcare resources such as hospital beds, ventilators, and medical staff in different scenarios. This information allows policymakers to allocate resources efficiently, ensuring that healthcare systems are adequately prepared to handle surges in cases. The model and the findings of the paper allow for the integration of both medical and non-medical interventions into the decision-making policy to prevent the virus spread. Thus, for example, social distancing and retail and recreation mobility percent change (as can be seen from Table 5) are the most important factors resulting in the total number of new cases and mortality ratio, while additional non-medical factors like temperature, immigrant students ratio, airport distance or housing density are among the most important features derived from the classification model (see Table 2). Hence, while developing the virus prevention (restriction) policy, the policymakers can consider such factors as the current and forecasted temperature, airport distance, and house density in the specific region etc., to restrict social distancing or retail or recreation closing or limitations.

Our work represents a significant advancement in classification ensemble methodologies. It offers a novel approach to cut-off determination that improves classification accuracy and adaptability in real-world applications. Future research will be related to using the developed ensembles for multimodal data analysis. Another possible approach is to use the imputed dataset, available in^21^. The authors used the KNN imputer to impute the missing values of a feature based on the other non-missing values of that feature for that county, with a few exceptions. However, in our opinion, this procedure makes the dataset to be an artificial one and not the real-world data. That’s why we do not examine the imputed dataset in this research. The comparison of the findings of this paper with the results of the machine-learning models applied to the imputed dataset will be carried out in future studies. Besides, other weak predictors could be used for ensembles as well as calibrated predictions of individual base models to ensure that their confidence estimates are well-calibrated and consistent across the ensemble. We plan to explore techniques for fusing the predictions of different models or datasets at various stages of the prediction process, such as feature fusion, decision fusion, or late fusion.

## Data Availability

The datasets generated and/or analysed during the current study are available in Figshare repository, https://figshare.com/articles/dataset/USA_covid-19_data/12986069/1.
