# Author Correction: A novel hybrid supervised and unsupervised hierarchical ensemble for COVID-19 cases and mortality prediction

**DOI:** 10.1038/s41598-024-66722-6

**Published:** 2024-07-08

**Authors:** Vitaliy Yakovyna, Nataliya Shakhovska, Aleksandra Szpakowska

**Affiliations:** 1 Faculty of Mathematics and Computer Science, University of Warmia and Mazury in Olsztyn, Ul. Oczapowskiego 2, 10‑719 Olsztyn, Poland; 2 Artificial Intelligence Department, Lviv Polytechnic National University, 12 S. Bandery St, Lviv, 79013 Ukraine; 3 Universytet Rolniczy, 31120 Kraków, Poland

Correction to: Scientific Reports, 10.1038/s41598-024-60637-y, published on 29 April 2024.

The original version of this Article contained an error.

In the section ‘Related works’,

"Alballa and Al-Turaiki^20^ address monetary policy concerning money laundering methods in COVID-19, focusing on diagnosis and predicting severity and mortality risk using machine learning algorithms.”

now reads:

"Alballa and Al-Turaiki^20^ focused on COVID-19 diagnosis and predicting severity and mortality risk using machine learning algorithms."

The original article has been corrected.

